# Voxel-Wise Feature Selection Method for CNN Binary Classification of Neuroimaging Data

**DOI:** 10.3389/fnins.2021.630747

**Published:** 2021-04-20

**Authors:** Domenico Messina, Pasquale Borrelli, Paolo Russo, Marco Salvatore, Marco Aiello

**Affiliations:** ^1^IRCCS SDN, Naples, Italy; ^2^Dipartimento di Fisica “Ettore Pancini”, Università Degli Studi di Napoli “Federico II” – Complesso Universitario di Monte Sant’Angelo, Naples, Italy

**Keywords:** deep learning, feature selection, neuroimaging, statistical parametric mapping, t-masking, Alzheimer’s disease, magnetic resonance imaging, brain disorders

## Abstract

Voxel-wise group analysis is presented as a novel feature selection (FS) technique for a deep learning (DL) approach to brain imaging data classification. The method, based on a voxel-wise two-sample *t*-test and denoted as *t*-masking, is integrated into the learning procedure as a data-driven FS strategy. t-Masking has been introduced in a convolutional neural network (CNN) for the test bench of binary classification of very-mild Alzheimer’s disease vs. normal control, using a structural magnetic resonance imaging dataset of 180 subjects. To better characterize the *t*-masking impact on CNN classification performance, six different experimental configurations were designed. Moreover, the performances of the presented FS method were compared to those of similar machine learning (ML) models that relied on different FS approaches. Overall, our results show an enhancement of about 6% in performance when *t*-masking was applied. Moreover, the reported performance enhancement was higher with respect to similar FS-based ML models. In addition, evaluation of the impact of *t*-masking on various selection rates has been provided, serving as a useful characterization for future insights. The proposed approach is also highly generalizable to other DL architectures, neuroimaging modalities, and brain pathologies.

## Introduction

During the last decade, technological advancements and the availability of large amounts of labeled data (Aiello et al., 2019) fostered neuroimaging research’s development (Traverso et al., 2020). In this context, machine learning (ML) algorithms played a relevant role (Lundervold and Lundervold, 2019; Chatterjee et al., 2020; Salmanpour, 2020; Traverso et al., 2020). Indeed, ML approaches aimed to automatically recognize meaningful patterns undetectable with human perception. ML methods enabled the potential development of computer-aided diagnosis and decision support systems for diagnosis and clinical management of a high number of diseases (Yassin et al., 2018). Moreover, ML algorithms were successfully applied to perform different tasks, like image classification, object detection, and image segmentation (Dora et al., 2017).

Concerning classification tasks, some ML studies focused on support vector machines (SVMs) and random forests for binary classification of pathological versus healthy conditions (Davatzikos, 2019) by using features manually extracted from raw data or features learned, in turn, by other simple ML models (Lundervold and Lundervold, 2019; Singh et al., 2020).

In the context of ML algorithms, deep learning (DL) gained considerable attention among the scientific community for medical imaging applications, hence also in the neuroimaging field (Ding et al., 2019; Jo et al., 2019; Spasov et al., 2019; Zhu et al., 2019). Unlike the classical ML approaches, where prior knowledge of the domain was fundamental, DL utilizes deep neural networks to automatically discover and extract useful features directly from the data (LeCun et al., 2015). Deep neural networks, in particular convolutional neural networks (CNNs), outperformed previous state-of-the-art ML approaches (Litjens et al., 2017; Shen et al., 2017; Brinker et al., 2019). In the context of neuroimaging, DL was applied to classify psychiatric and neurological disorders, which tend to be associated with subtle and diffuse neuroanatomical and neurofunctional abnormalities (Vieira et al., 2017).

Typically, ML approaches widely used feature selection (FS) techniques, i.e., the process of choosing a subset of relevant features for use in model design. The FS goal is finding the best feature subset that yields the minimum generalization error, enhancing the model’s performance (Vergara and Estevez, 2014). FS methods improve the generalization by avoiding the curse of dimensionality and by reducing overfitting (Singh et al., 2016). Furthermore, FS techniques are increasingly employed since they allow a simplification of learning models, facilitating human interpretability. They also have the advantage of diminishing training times (Venkatesh and Anuradha, 2019). However, these ML approaches share the requirement of high domain technical knowledge, thus limiting their applicability to specific classification tasks (LeCun et al., 2015). Data-driven approaches provided fundamental improvements for the generalization of ML algorithms to deal with this issue. For example, Ijaz et al. (2020) utilized a data-driven prediction model with outlier detection based on random forests and used a chi-squared FS method. Instead, Ijaz et al. (2018) uses an *Information Gain* technique to evaluate the features’ significance.

In general, DL algorithms did not require FS approaches since DL automatically discovers the intricate structure of imaging features in large datasets during the training step (LeCun et al., 2015). However, FS techniques could improve the DL performance for a classification task, mostly in the case of limited data availability (Chen et al., 2020; Raj et al., 2020). Indeed, in the domain of computational neuroscience, the available image datasets are often characterized by the size of features much larger than the number of examples (the curse of dimensionality) (Abraham et al., 2014; Mwangi et al., 2014). In the context of high-dimensional classification, in general, FS approaches have been proven suitable in several biomedical applications, also in DL employments (Fan and Fan, 2008; Suk et al., 2016; Raj et al., 2020). However, in deep neural network models, FS methods are still poorly investigated (Raj et al., 2020).

Voxel-wise two-sample *t*-tests were demonstrated to be effective in revealing brain areas associated with statistically significant differences between groups related to a multitude of neurological pathologies, for example, Alzheimer’s disease (AD) (Senjem et al., 2005), multiple sclerosis (Audoin et al., 2010), amyotrophic lateral sclerosis (Sage et al., 2007), childhood absence epilepsy (Pardoe et al., 2008), and schizophrenia (Asami et al., 2014).

To date, limited attention has been paid to deriving statistical significance maps of imaging patterns to establish if a voxel or a deep feature constitutes a significant contributor to a DL model (Davatzikos, 2019). In general, FS approaches based on statistical saliency are introduced in association with simpler classifiers than those in DL models. For example, in Tohka et al. (2016), several data-driven FS and classification methods are proposed for the whole-brain voxel-based classification of AD vs. normal control (NC) subjects.

The inclusion of an FS criterion into a DL pipeline represents an attractive and non-trivial challenge due to the non-linear nature of DL models. Moreover, this criterion should be automatic, generalizable, and data driven, to maintain the critical aspect of DL: to have multiple layers of features not designed by human engineers, but learned from data using a general-purpose learning procedure (LeCun et al., 2015).

In this work, we introduced a novel FS technique for CNN architectures. Our primary aim was to verify the effective improvement of a binary classification task by including a data-driven FS approach based on a voxel-wise test of statistical significance. The choice of applying FS to a CNN algorithm was motivated by the reduction of the sample dimensionality without losing relevant information. Indeed, the image dimensionality (e.g., the number of voxels) represents a bottleneck that affects the CNN routines’ training for 3D medical imaging classification procedures (Vieira et al., 2017). We aimed to demonstrate the *t*-masking approach’s feasibility by assessing its impact on a 3D CNN model’s performance. For this purpose, we choose, as a test bench, the classification task of AD vs. NC subjects, widely discussed in the literature. The main reasons for this choice were as follows: (i) It is demonstrated that CNNs have a good baseline level of accuracy performance for datasets of a few hundreds of samples, also in the early stage of pathology (Vieira et al., 2017) and (ii) it is demonstrated that there are subtle region-specific anatomical alterations in AD brains, viewable via structural T1-weighted (T1-w) magnetic resonance imaging (MRI) (Fox and Freeborough, 1997; Ling et al., 2013) and, likely, detectable by a voxel-wise *t*-test. In particular, the classification task consisted of automatically recognizing subjects with very mild AD vs. NC, a task more challenging compared to the AD-vs.-NC classification. However, the task under consideration has a higher clinical interest for early diagnosis development.

In the AD-vs.-NC classification task context, a massive quantity of studies based on ML and DL methods was published. Early studies on AD classification proposed a classification pipeline that started with an FS and extraction steps to obtain useful information to feed on a multivariate pattern classification algorithm (Mateos-Pérez et al., 2018). One of the most employed classifiers was SVM, requiring kernels that transform input data and act as a similarity measure for the classification task. Referring to the FS strategy, Chaves et al. (2009) used an FS based on a *t*-test to select regions of interest, reducing the dimensionality of input data; Asim et al. (2018) utilized a brain atlas; Moradi et al. (2015) used a simpler ML algorithm as regularized logistic regression; Suk et al. (2016) proposed a weighted sparse multi-task learning method, and Zhang et al. (2014) adopted a principal component analysis (PCA) method. Fan et al. (2007) performed a combination of group analysis on adaptive regional elements with an SVM classifier. Ensemble methods (like the random forest) are also employed for the multimodal classification of AD (Gray et al., 2013).

DL methods have been applied to AD-vs.-NC classification tasks, achieving the highest classification performance, especially when using multimodality data or when combined with other learning approaches (Jo et al., 2019). Spasov et al. (2019) developed a parameter-efficient 3D CNN with a dual-learning approach to predict the conversion from mild cognitive impairment (MCI) to AD. Khagi et al. (2019) used transfer learning to enhance the performance of their CNN. Afzal et al. (2019) proposed an augmentation technique to balance the dataset and improve classification performance, increasing the training set’s sample size. Suk et al. (2016) presented a DL-based latent feature representation with a stacked autoencoder, while Choi et al. (2018) introduced PET images as input in a 3D CNN to predict conversion from MCI to AD.

Considering the analyzed literature, to the best of our knowledge, this is the first study in the neuroimaging field developing a DL model with an FS based on voxel-wise statistical analysis for image binary classification.

## Materials and Methods

### Dataset

Data from the OASIS-3 release, which consisted of a longitudinal neuroimaging, clinical, and cognitive study of normal aging and AD (LaMontagne et al., 2019), were used. In particular, we selected T1-w MRI data acquired with a 3-T magnetic resonance (MR) scanner. LaMontagne et al. (2019) reported detailed information on recruitment criteria, imaging acquisition protocols, used scanners, and clinical/neurological assessment. We used the Clinical Dementia Rating (CDR), as provided by OASIS-3, to select AD subjects with very mild AD (i.e., CDR = 0.5). We selected structural T1-w MR images from 90 AD subjects with an MR session made at the time close to AD diagnosis with CDR = 0.5. To obtain a balanced dataset of both AD and NC groups, 90 NC participants, matched for age and sex with the selected AD group, were randomly included by selecting the T1-w images related to the first available sessions, resulting in a dataset with 180 samples.

### Preprocessing

The preprocessing procedures consisted of spatial and intensity normalization of neuroimaging data.

Spatial normalization was performed by registering the T1-w images to the Montreal National Institute (MNI)-152 (1 mm) standard space template using the FSL routines (FMRIB-FSL package v. 6.0.0). In particular, we used the FNIRT routine to non-linearly register the T1-w images to the MNI-152 template, including a preliminary linear registration step (FLIRT of FMRIB-FSL) of the brain-extracted T1-w images. The skull stripped T1-w images were generated with a 3D CNN approach, using the DeepBrain Extractor, a Python tool that runs a pre-trained 3D U-net available at https://github/iitzco/deepbrain. The spatial normalization results were visually checked to assess the quality of the normalization procedure (i.e., anatomical consistency among the registered images).

The intensity normalization procedure consisted of rescaling the voxel values to zero mean and unit standard deviation, making it easier to learn the weights to the optimization algorithm (Raschka and Mirjalili, 2017).

We obtained two preprocessed datasets, detailed as follows, by applying different combinations of spatial and intensity normalization procedures:

• T1-w images processed for intensity normalization (denoted as raw T1-w images) and
• T1-w image obtained by applying spatial and intensity normalization routines (denoted as spatially normalized T1-w images).

Subsequently, we randomly separated the dataset into three subsets: a training set (108 subjects, 60% of the dataset), a validation set (36 subjects, 20% of the dataset), and a test set (36 subjects, 20% of the dataset). We paid particular attention to obtaining the same number of samples per class (i.e., NC and AD) in each subset to get a balanced training dataset.

### t-Masking CNN Model

Let *X* be the training dataset of spatially normalized brain images with 2*N* examples; for a given binary classification task, we define *X*_0_ as the subset of 0-labeled images and *X*_1_ as the subset of 1-labeled images. *X* was built as a balanced dataset; i.e., *X*_0_ and *X*_1_ have the same size *N*. Let *X*_0_*_*j*_*(*υ*) be the intensity value of the *j*th image in *X*_0_ at the voxel *υ*. The voxel intensity sets {*X*_0_*_*j*_*(*υ*) | ∀ *j* ∈ [1, *N*]} and {*X*_1_*_*j*_*(*υ*) | ∀ *j* ∈ [1, *N*]} are assumed to refer to the same brain location for all elements since the images are spatially normalized. Let *μ*_0_(*υ*) and *μ*_1_(*υ*) be the means for the two precedent sets, respectively; hence, we recall the definition of the Student *t*-statistic (Snedecor and Cochran, 1989), to define the *t*-map in terms of the *t*-score:

(1) $$t\left(v\right)=\frac{\left|\mu_{0}\left(v\right)-\mu_{1}\left(v\right)\right|}{\sqrt{0.5\left(\sigma_{0}^{2}\left(v\right)+\sigma_{1}^{2}\left(v\right)\right)}}$$

where *σ*_0_ and *σ*_1_ are the standard deviations for *X*_0_*_*j*_*(*υ*) and *X*_1_*_*k*_*(*υ*) for all *j* and *k* in [1, … *N*], respectively. Considering the null hypothesis *H*_0_: *μ*_0_ = *μ*_1_, we reject *H*_0_ if *t*(*v*) > threshold. Repeating the computation for all *v*, we obtain a multiple-hypothesis test-based map, namely, the *t*-mask, corresponding to voxels belonging to brain areas with statistically different values between *μ*_0_ and *μ*_1_, at a given threshold. As a result, only the most salient voxels for the given binary classification task will be considered as relevant features with the *t*-masking application. Features with 0-value in the *t*-mask were considered as redundant features, according to the definition of Vergara and Estevez (2014).

The *t*-masking approach was included in a CNN model. Below, we briefly describe the CNN architecture.

Convolutional layers in a 3D CNN are composed of *K* different filters. Each filter works by convolving an input tensor *x* with a 3D kernel of weights *W* ∈ *R*^*m* × *m* × *m*^ with size *M* = *m*^3^ and adding a bias term *b*. The result is passed to a non-linear activation function *f*(⋅). Therefore, each filter *k* returns a feature map *h*_*k*_, extracting a derivative of *x*, as follows:

(2) $$h_{k}=f\left(W_{k}*x+b_{k}\right)$$

The set of *K* feature maps, extracted from the input *x*, defines a single layer ℓ in a CNN architecture composed of *L* layers. The *k*th feature map at layer ℓ, denoted as *h^*l*^_*k*_*, is constructed using the outputs of layer ℓ - 1 as inputs to layer ℓ:

(3) $$h_{k}^{l}=f\left(W_{k}^{l}*h^{l-1}+b_{k}^{l}\right)$$

For simplicity, we neglect the description of the pooling layers, which, although fundamental for the CNN model, are not relevant for our purpose. Therefore, the last layer’s output *θ* is inputted in a cross-entropy cost function *J*(*θ*), which depends on a chain of the precedent layers’ output (Eq. 3). After a random initialization of the weights, the network is trained in order to update the vector of all weights *W* and *b* with gradient-based optimization, using the well-known backpropagation algorithm.

t-Masking induces a simplification of the complex structure of *J*(*θ*). To explain this, we consider the affine transformations of the first layer as

(4) $$W_{k}^{0}*x+b_{k}^{0}$$

Eq. (4) could be dominated by redundant voxel intensity values. Indeed, Eq. (4) could be reformulated as

(5) $$W_{k}^{0}*\left(x^{rel}+x^{red}\right)+b_{k}^{0}$$

where *x*^*rel*^ and *x*^*red*^ represent the matrix of relevant and redundant voxel values, respectively, associable to *w*_*i*_ with an appropriate factorization. When ||*x*^*rel*^||_1_ ≫ ||*x*^*red*^||_1_, the affine transformations are dominated by the redundant terms, principally characterized by low-ranking *t*-scores. Therefore, it should produce a noise-like effect in deeper layers due to relevant patterns hidden by indistinguishable redundant terms. t-Masking imposes *x*^*red*^ = 0 for all *i*, improving filters’ response when complex interactions in deep layers between *x*^*rel*^ and *x*^*red*^ can be neglected. Furthermore, it is well known that CNNs have sparse interactions between input features in deep layers (Goodfellow et al., 2017), as evident from Eq. (3). Therefore, considering that *t*-masking preserves the spatial relationship of data, its application to a CNN allows sparse interactions among all relevant voxels (*x_*ij*_^*rel*^*, thus serving the requirements of a CNN. Consequently, the model can extract meaningful information just from the relevant voxels’ spatial distribution. This could be an advantage over applying *t*-masking to a classical ML model, which generally requires the vectorization of the input features.

In conclusion, such formal description highlights how *t*-masking can minimize the redundancy, reducing the features’ dimensionality, thus removing possible bias sources in the classification task.

### Feature Selection

**FIGURE 1:**
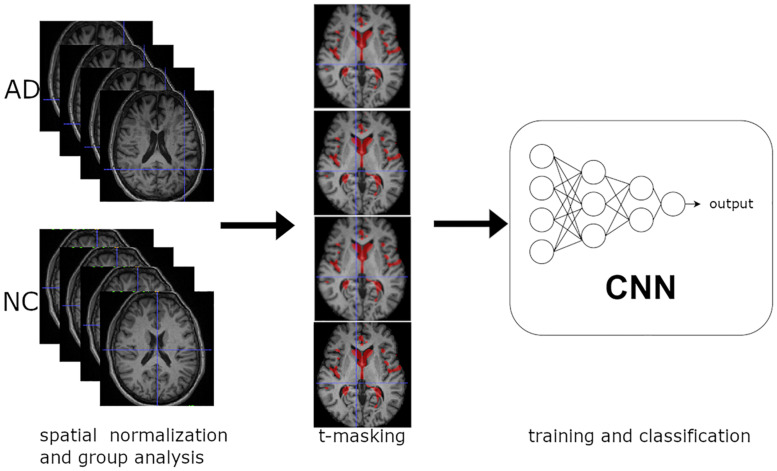
Overview of the learning procedure: (1) Spatial normalization of MR structural images and group analysis on training data. (2) *t*-Masking of input images. (3) Training and classification of 3D CNN.

Figure 1 shows a synthetic scheme of the learning procedure. We performed a voxel-wise two-sample *t*-test between spatially normalized T1-w images from NC and very mild AD, with Gaussian smoothing with a full width at half maximum of 6 mm. We applied the *t*-masking to all data (training, validation, and test set) as a binary mask (with a fixed threshold).

We computed the *t*-map, *t*(*v*) only on the training set to avoid leakage, i.e., the creation and usage of variables (like labels in the case of classification tasks) that carry information about the outcome of the classification task (Mateos-Pérez et al., 2018). The choice of a suitable threshold for the previous step was investigated, evaluating the classification performance at variable *t*-masking threshold through validation curves. We used the accuracy and the area under the curve (AUC) of receiver operating characteristic (ROC) as metrics for the plots. Train, validation, and test accuracy, along with validation and test AUC curves, are plotted (Figure 4) to give a qualitative assessment of the bias, variance, and the model’s stability as the threshold changes.

### CNN Architecture Overview

We utilized a 3D CNN inspired by the study of Spasov et al. (2019) that already proposed an efficient CNN model (about 5,105 parameters) with high accuracy for AD/MCI conversion prediction. They implemented a dual-learning approach combining several input streams such as structural MRI measures, Jacobian determinant images, and clinical data. We modified such architecture by simplifying and tailoring it for our purposes. In particular, with a Siamese approach, input data were kept only from MRI, eliminating clinical data input, their associated subnetwork, and Jacobian determinant images. Moreover, we removed the dual-learning method, preserving a logistic regression as a binary classifier. The network was designed to receive input masked MR images on two parallel layers. After two layers, the outputs were concatenated, merging the activation maps along the channel axis (the concatenate layer). The add-block performed element-wise addition between two sets of activation maps of the same size along all dimensions. A residual connection in the add-block facilitated training in analogy to ResNet behavior. The network decreased the image inputs’ dimensionality using standard, separable, and grouped convolutional blocks before two fully connected layers. Its output was a four-dimensional feature vector, ready for the logistic regression classifier. The following network settings remained unchanged in all experiments: (i) the dropout rate, set at 0.1 for all layers and blocks; (ii) the L2 regularization penalty coefficient, set at 5 × 10^–5^ for all parameters in the convolutional and fully connected layers. The convolutional kernel weight initialization followed the procedure described by He et al. (2015). The objective function loss was minimized using the Adam optimizer (Kingma and Ba, 2017), with an exponentially decaying learning rate of 0.001 × 0.3 epoch/10. A training batch size of six samples was randomly sampled from the dataset when training the network until the dataset was exhausted.

**FIGURE 2:**
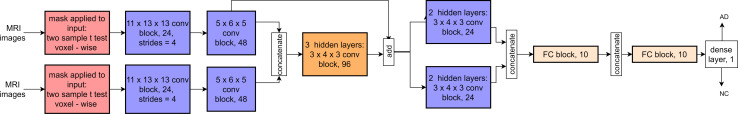
The architecture of our 3D CNN with a Siamese approach. The notation of blocks is as follows: kernel size, (separable) convolutional block, and output channels. If the strides are different from the default value of 1, the new stride value is shown in addition at the end. Each (separable) convolutional block sequentially contains a 3D (separable) convolutional layer, batch normalization, ELU activation, 3D max-pooling (only the convolutional layer), and dropout. FC blocks contain a fully connected layer, batch normalization, ELU activation, and dropout. For details, see Spasov et al. (2019).

A synthetic scheme showing the adopted CNN architecture is presented in Figure 2.

#### Regularization and Hyperparameter Choice

We adopted several strategies to manage overfitting, some of which were in common with the Spasov model (batch normalization, dropout, and L2 regularization). Since the previous work of Spasov already optimized the hyperparameters like batch size, learning rate, dropout rate, and the L2 regularization parameter, we used the proposed configuration for those parameters. To control the overfitting and reduce computational time, a further implicit regularization, the early stopping (Yao et al., 2007), was included. It consists of stopping the training of the network before it ceases to improve generalization performance. In particular, we imposed the patience parameter at 10 epochs; i.e., we retrieved the model with the minimum validation loss if the last 10 epochs did not obtain a lower validation loss.

### Experimental Models

To assess the classification performance of the proposed CNN model, we implemented seven different experimental configurations, as detailed in the following points:

1. 3D CNN model, without FS, on raw T1-w images (raw_MRI).
2. The same model as point 1 to spatially normalized T1-w images (norm_MRI).
3. 3D CNN model with *t*-masking FS (fs_CNN_MRI).
4. A linear classifier with an FS based on *t*-masking (fs_linear_MRI).
5. 3D CNN model with *t*-masking FS corrupted by adding Gaussian noise with 0 mean and 0.2 standard deviation (fs_noise_CNN_MRI).
6. 3D CNN model with *t*-masking FS biased by randomly turning off voxels in the *t*-mask, following a Bernoulli probability distribution with *p* = 0.05 (fs_bernoulli_CNN_MRI).
7. 3D CNN model with a random FS based on a “voxel-wise” dropout that turns off the voxels with a constant Bernoulli distribution probability *p*. A validation curve was performed as a function of *p* ranging from 0.01 to 0.99 (fs_random_CNN_MRI).

We implemented fs_linear_MRI to verify the classification performance of the voxel-wise two-sample *t*-test without the CNN. In that experimental setup, we replaced the CNN with a linear classifier that receives in input the mean of the masked image voxel values. The application of *t*-masking to the linear decision model (classification of average intensity voxels by a perceptron) provided an estimate of the statistical mapping contribution alone.

The fs_noise_CNN_MRI and fs_bernoulli_CNN_MRI processes allowed us to assess the robustness of fs_CNN_MRI to small variations of selected voxels by perturbing the *t*-mask. fs_noise_CNN_MRI was thought to assess the *t*-masking’s robustness to structural MRI misalignment and subjects’ misclassifications. fs_bernoulli_CNN_MRI was planned to evaluate the robustness to type I and II error variations. We implemented fs_random_CNN_MRI to compare *t*-masking applied to the same CNN using another FS technique.

Each experimental point was obtained by averaging metrics acquired from five random (i.e., with a random seed) sample permutations. Raw_MRI and norm_MRI correspond to a single experimental point. In all other cases, we evaluated a validation curve from the applied experimental points to study the different behaviors at different threshold values of the *t*-map. In detail, for models from 3 to 6, we evaluated 21 experimental points, corresponding to several threshold points related to the binary mask obtained from the *t*-map. The threshold values were selected in the equally spaced range [0, 8] with a zero-threshold corresponding to the entire brain mask and an eight-threshold representing no surviving voxels in the binary mask.

### Performance Evaluation

We compared the computed models’ performance metrics (from model 3. to 6. listed above) through validation curves as a function of the *t*-mask threshold. Test accuracy and ROC analysis were executed by computing the AUC. We adopted a dedicated cross-validation method to determine the quantitative effect of random sampling for splitting the dataset on the training process. In particular, we tested whether the FS method outperformed the baseline CNN by considering the average between each permutation of test accuracy and test AUC, named the average cross-validation. Instead, in the classical cross-validation, the performance was evaluated by observing test accuracy/AUC corresponding to the model with the highest value of the metrics on the validation set. This method allowed us to identify what thresholds outperform baseline models, and it enabled us to compare the validation curves of all our models in a statistically meaningful way. Moreover, to allow a further comparison with other literature methods, we added the test accuracy and the test AUC obtained by the classically fivefold cross-validation; i.e., we selected the model which achieves the best performance on the validation set.

We evaluated the performance enhancement (PE) of *t*-masking by comparing the peak accuracy of fs_CNN_MRI with the accuracy of the raw_MRI model. In particular, PE is computed as the difference of the test accuracy of two models, estimated with average cross-validation; negative PE indicates that the considered model underperforms the reference model. We also compared the PE result with the PE of typical FS approaches compared on a previous work (Tohka et al., 2016). These models are trained on 200 subjects from ADNI MRI T1-w to resolve an AD-vs.-NC classification task (with no CDR subjects’ selection).

Furthermore, we included a visual explanation method as a qualitative performance evaluation, based on the computation of a saliency map (heat-map) that localizes relevant image regions for the CNN model, resulting in a saliency map of features. In particular, we implemented a class activation mapping (CAM) method for 3D CNNs, by using the grad-CAM algorithm developed by Selvaraju et al. (2020). The heat-map was derived by computing the gradient between a convolutional layer output and the loss output. We inspected the output of the first “concatenate” layer (Figure 2), as a compromise between the deepest layer possible for maintaining high-level semantics and the highest map resolution (Selvaraju et al., 2020). We applied grad-CAM to the models derived from each considered threshold of fs_CNN_MRI and norm_MRI.

### Algorithm Implementation

All experiments were conducted using Python 3.6.9. The neural network was built with the Keras DL library using TensorFlow 2.0 as a backend. 3D convolutions were available as a Keras module. The 3D separable and grouped convolutions modules were available in previous works (Spasov et al., 2019). The code, available to https://github.com/simeon-spasov/MCI, was adapted for the v.2 of TensorFlow^1^ and v.3 of Python. To compute the *t*-map, we employed the Python module nistats, included in nilearn (“Nistats: Functional MRI in Python—functional MRI for NeuroImaging,” n.d.). For the whole experimental procedure, we utilized a cloud computing strategy: a free Jupiter notebook environment working directly on a browser with a virtual machine that requires no setup to use while providing free access to computing resources, including GPU (up to 25.51-GB RAM, a GPU T4 or P100). The code to replicate the experiments is available at https://gitlab.com/sdndeep/voxel_wise_fs.

Employing the resources available for Colab users (Colab offers only one core CPU), we run *t*-masking with limited computational power. Therefore, currently available workstations (or PCs) on the market could obtain better time performances, considering a faster charge dataset on RAM and the statistical test’s parallelizability.

It is important to underline that the addition of *t*-masking provides an additional computational burden which may not be negligible when large datasets are used. Indeed, it should be considered that to perform the two-sample *t*-test, the entire training set must be loaded in RAM memory to compute the *t*-mask.

## Results

**FIGURE 3:**
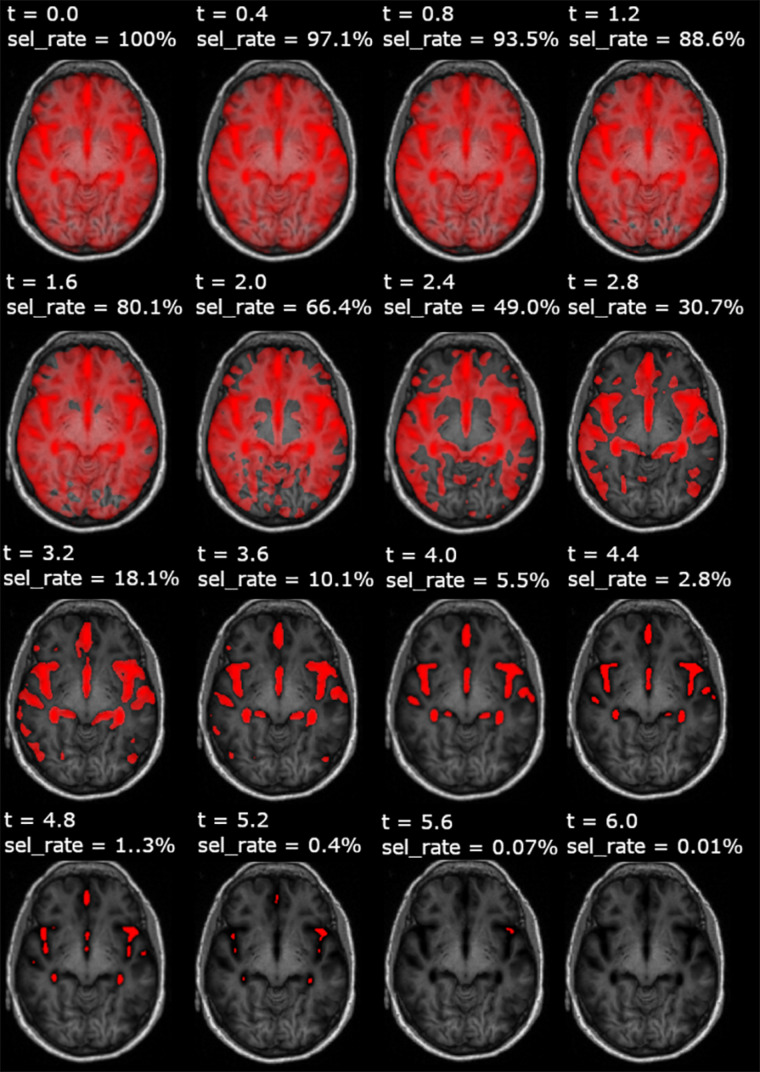
Feature selection maps (*t*-masking) overlaid on a spatially normalized axial T1-w images, for each adopted threshold (*t*). This figure represents an example resulting from a *t*-masking pipeline run applied to very mild AD- vs. -NC classification. For each *t*-masking, the fraction of voxels selected for a given threshold with respect to the whole-brain volume is also reported (sel_rate).

In Figure 3, we show an example of *t*-masks, obtained within a fs_CNN_MRI training run, at different thresholds of *t*-parameter, in the range [0.0, 6.0].

Table 1 shows the results of the tested experimental models, in terms of both accuracy and AUC.

**TABLE 1:** Test accuracy and AUC evaluated for each tested method with average cross-validation (the average between permutations) and classical cross-validation (the highest between permutations).

Experimental models	Average cross-validation		Classical cross-validation	
	Test accuracy	Test AUC	Test accuracy	Test AUC
raw_MRI	(69 ± 2)%	0.79 ± 0.01	78%	0.82
norm_MRI	(68 ± 2)%	0.80 ± 0.01	78%	0.83
**fs_CNN_MRI (*t* = 3.6)**	(75 ± 1)%	0.85 ± 0.01	81%	0.88
fs_linear_MRI (*t* = 4.4)	(69 ± 2)%	0.78 ± 0.02	72%	0.81
fs_noise_CNN_MRI (*t* = 4.4)	(76 ± 3)%	0.84 ± 0.03	72%	0.81
fs_bernoulli_CNN_MRI (*t* = 4.4)	(74 ± 3)%	0.82 ± 0.03	69%	0.83
fs_random_CNN_MRI (*p* = 71%)	(57 ± 8)%	0.62 ± 0.15	64%	0.69

*Please refer to the text for the details of the tested methods.*

Overall, the fs_CNN_MRI model holds the highest performance for both average and classical cross-validation metrics. In particular, considering the average cross-validation metric, the fs_CNN_MRI outperforms the raw_MRI and norm_MRI models with PE values of (6 ± 2)% and (7 ± 2)% (accuracy) and differences in AUC of 0.06 ± 0.02 and 0.05 ± 0.02, respectively. The results were also confirmed for classical cross-validation metrics, where fs_CNN_MRI outperforms both the raw_MRI (PE: 3%; AUC difference: 0.06) and norm_MRI (PE: 3%; AUC difference: 0.05).

**FIGURE 4:**
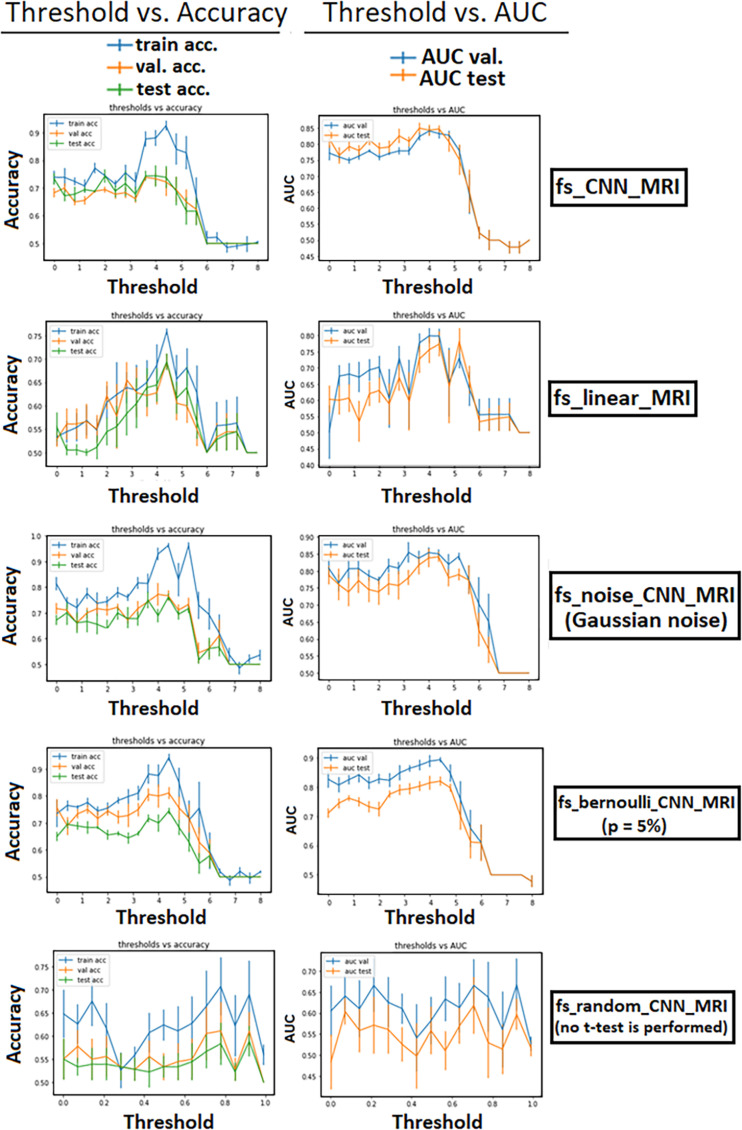
Performance of the models as a function of the *t*-mask threshold. Error bars represent the standard deviation of the mean. For all *t*-masking models, the curves show how the metrics reach a peak value, improving the performance to the threshold increase until a final breakdown due to the loss of relevant voxels. The zero-point thresholds are equivalent measures of the norm_MRI model for fs_CNN_MRI and fs_noise_CNN_MRI. The bottom row shows the results in the case of “voxel-wise” dropout (with increasing *p*-value on the abscissa). There is evidence that the performance of fs_random_CNN_MRI rapidly decreases at increasing *p*-value.

Figure 4 shows the *t*-mask threshold setting’s influence on the performance of the CNN models in terms of validation curves for all metrics (train–validation–test accuracy, validation, and test AUC). We observe that the average performance increases until reaching a peak in all models utilizing the FS method. For all *t*-masking models, the curves show how the metrics reach a peak value, improving the performance to the threshold increase until a final breakdown due to the loss of relevant voxels. The peak is in the same threshold range [3, 5] for all models, which means, recalling Figure 3, the percentage of selected voxels is between approximately 18% and 0.4%.

We evaluated the PE of *t*-masking using average cross-validation results for test accuracy between fs_CNN_MRI and raw_MRI. We obtained a PE of (6 ± 2)%, as shown in Table 1. Table 2 shows a comparison of our experimental PE results with a precedent work in the literature (Tohka et al., 2016). The original article’s notation is maintained. In this paper, the authors studied an AD-vs.-NC classification task, applying several FS techniques to classical ML classifiers. They compared SVM, with or without filter-based FS; several embedded FS methods; and stability selection (with logistic regression, lasso, elastic net, and graph net). Filter-based methods consisted of a *t*-test-based filter, with or without a false discovery rate (FDR)-corrected threshold. Their classification task is not directly comparable since they used the ADNI dataset without a CDR selection.

**TABLE 2:** Comparison of PE for models using FS techniques, analyzed in Tohka et al. (2016).

	Performance enhancement
EN-VACV	+1.8%
EN-VABEE	+1.4%
EN-05CV	+1.8%
EN-05BEE	+1.0%
LASSOCV	+1.4%
LASSOBEE	0%
LASSOSTAB	−0.2%
EN-05STAB	−0.1%
GNCV	+1.5%
GNBEE	+0.7%
SVMF-FDR	+1.3%
SVMF-1000	+1.6%
SVMF-125	+2.1%
SVM-ALL	0%
***t*-Masking CNN**	**+6 ± 2%**

*The original article’s notation is maintained. The results are referred to an average of the experimental conditions with resampled images to 4 and 8 mm isotropic and spatial resolutions. In the case of results from Tohka et al. (2016), the PE is computed by subtracting the test accuracy of the model under consideration with SVM-ALL test accuracy; in the case of fs_CNN_MRI, the PE is computed by a subtraction with raw_MRI. Negative values refer to worse performance.*

Finally, to provide the reader with an idea of the operational feasibility of the method, we evaluated the additional computational time required by *t*-masking; it took about 5 min for the training dataset (108 subjects), considering the experimental setup described above.

The heat-maps derived from the grad-CAM algorithm, at varying *t*-thresholds for the fs_CNN_MRI experiment, are reported in Supplementary Figure 1. It is clear, from the heat-maps, that the greater the weight of the *t*-masking, the greater the focus of the grad-CAM model on the salient regions of interest for the classification task.

## Discussion

We proposed a *t*-masking FS method for deep neural networks on a CNN architecture by evaluating its performance and the behavior with respect to the adopted salience parameter (*t*-threshold). We tested the proposed CNN model for a very mild AD-vs.-NC classification task with an structural MRI dataset of 180 subjects. It is the first time a feature selection technique is investigated for DL applications to neuroimaging, to the best of our knowledge. By comparing raw_MRI and norm_MRI models with fs_CNN_MRI, we can affirm that the adoption of the *t*-masking resulted in an effective increase in performance. In particular, it should be noted that raw_MRI and norm_MRI reached similar performance. For this reason, we can deduce that the spatial normalization process did not degrade the classification performance and, notably, that the enhancement of fs_CNN_MRI can be reasonably attributed solely to the *t*-masking.

Moreover, the similar performance between fs_linear_MRI (which applies *t*-masking to a linear model) and both raw_MRI and norm_MRI indicated that the classification based only on statistical mapping achieved similar results compared with using CNN alone, whereas it reached worse results compared with fs_CNN_MRI. We showed that in fs_noise_CNN_MRI and fs_bernoulli_CNN_MRI, the overfitting slightly increases. We should also note that validation curves verified the increasing linear dependence of the performance as a function of the selected threshold (for models from 3. to 6.). It is interesting that the PE peak was reached in the same threshold range [3, 5] for all *t*-masking models. This observation can be interpreted as *t*-masking results being robust to small attempts to mess up the *t*-map, despite a slight increase in overfitting and inferior generalization capability. Finally, fs_random_CNN_MRI model results showed that the performance, in terms of both accuracy and AUC, decreased if a random voxel-wise FS replaces the *t*-mask. This comparison is a further confirmation of how *t*-masking selected relevant features against the random FS approach. As shown in Figures 3, 4, relevant features, i.e., features leading to better classification performance, represented a small 10.1% fraction of the total brain, thus confirming the working hypothesis explained in Section 2.3 (||*x*^*rel*^||_1_ ≫ ||*x*^*red*^||_1_). Furthermore, grad-CAM results show that *t*-masking contributes to the focalization of relevant image regions for the model training with respect to norm_MRI (*t* = 0.0), which instead shows a more dispersive feature distribution. As expected, as the *t*-threshold grows, the heat-maps highlight more focused regions for the classification task.

We analyzed early AD vs. NC, as a test bench, for a demonstrated difference between AD brains and NC brains, due to the presence of subtle region-specific anatomical alterations in AD brains, viewable via structural MRI T1-w and detectable by a voxel-wise *t*-test. The obtained results justify this choice and allow us to deduce that the same method could be applied to disorders with the same characteristics.

In the method here presented, we have chosen a voxel-wise two-sample *t*-test, considering that it is a standard method for mass univariate analysis and represents a well-established statistical tool for group analysis in the neuroimaging field. In principle, other statistical tests, such as mass multivariate methods, could serve as suitable FS methods and deserve further investigation. Indeed, considering Figure 3, other statistical maps could obtain a similar distribution of relevant voxels.

In this work, the CNN architecture proposed by Spasov et al. (2019) was adopted due to both its high performance and efficiency in the AD-vs.-NC classification task. However, different CNN architectures could be used, and further investigations are required to assess the *t*-masking performance when different CNN architectures are adopted.

We obtained the best PE with a reduction of 90% brain voxels, protecting from the curse of dimensionality. However, in the DL literature, FS techniques are often under-investigated since deep neural networks aim to implicitly extract the relevant features. Based on our findings, we argued that FS techniques, like *t*-masking, should deserve more attention in DL models’ design. The *t*-masking approach is integrated into the learning process and is based only on simple statistical analysis on training data, with limited *a priori* hypotheses. For this reason, it is in line with the fundamental ideas of DL.

It is possible to adopt data augmentation, pre-trained layers, or transfer learning strategies to obtain better performance. Transfer learning reaches state-of-the-art performance in pathological brain detection with AlexNet-based neural network architectures, as demonstrated by Lu et al. (2020) and Lu et al. (2019). We excluded the application of these techniques since this work was focused just on the impact of FS on the performance. However, the study of the impact of the concurrent combination of multiple strategies to the global performance of a DL model deserves attention and further investigations.

In the context of the CDR-based AD-vs.-NC classification task with transfer learning, Nanni et al. (2020) reached 0.83 AUC when only very mild AD subjects (CDR = 0.5) were selected. With a 0.88 test AUC, the proposed *t*-masking model fs_CNN_MRI outperformed the ensemble transfer learning model for the same classification task and reached comparable performance when a conventional ML was applied to the same classification task (0.89 AUC).

Our results show that *t*-masking could not improve the performance under some specific conditions. Indeed, we have found that when the number of selected voxels was limited, the performance deteriorated with increasing overfitting. This result could be ascribed to the loss of relevant information when decreasing the number of voxels. If *t*-masking is considered for different classification tasks, the active regions (i.e., regions in which voxels are selected by masking) might be too few. A high variability as a function of the training set could be found, depending on the brain disorder’s intrinsic physiology. A possible solution could be to use different imaging modalities or to exploit multimodal imaging. A further limitation of our study is represented by the dataset size, and further investigations are required to test how our FS technique improves the classification performance capabilities on a larger dataset.

The FS *t*-masking approach deserves attention also for the possibility of being easily generalizable for the classification of other pathologies, as well as for other neuroimaging modalities (if it is possible to make a voxel-wise comparison through a spatial normalization). Furthermore, the *t*-masking, although demonstrated on a CNN in this work, can, in principle, be used to perform FS in other DL architectures.

Considering the comparison with results obtained by Tohka et al. (2016), as reported in Table 2, it should be noted that their experimental procedure differed from ours due to the use of a split-half resampling-type analysis. However, they reported an average test accuracy. For this reason, average cross-validation results are more suitable for comparison than classical cross-validation, despite the latter method being more used in the literature. Despite the less challenging classification task (AD vs. NC with respect to very mild AD vs. NC, in our work) and the differences in the analyzed dataset (for example, MRI T1-w images acquired with a 1.5-T scanner), it is interesting to compare their results with ours by using the PE as a more objective comparison criterion. In particular, we have achieved a threefold improvement in performance compared to their algorithms. It is useful to underline that the model SVM-ALL refers to an SVM classifier without any FS application and, consequently, that it is possible to compare the PE obtained by Tohka et al. (2016) by using FS methods. Accuracy comparison cannot be applied due to the different data sources. Moreover, the presented comparison should be analyzed considering that they did not select AD subjects using a CDR-based analysis. We did not find other studies analyzing a very mild AD-vs.-NC binary classification task on the OASIS-3 dataset. However, Saraswathi et al. (2013) used a GA-ELM-PSO (refer to the article for the notation) classifier for AD multiclass classification by CDR-level voxel-based morphometry for feature extraction and a genetic algorithm for FS. They used an OASIS dataset (not OASIS-3), obtaining an 81% test accuracy for the very mild classification. In real-world applications, *t*-masking could be suitable in contexts with hundreds (or few thousands) of subjects available for training. The use of *t*-masking indeed requires increased use of resources during training. In particular, the computation of the *t*-mask requires an additional computational step in the training phase, thus requiring proper RAM resources and extra computational time. Therefore, the *t*-masking CNN model pays the performance improvement with a higher training time than a CNN alone because *t*-masking takes about 5 min to compute the *t*-mask of 108 subjects. Besides, if properly managed, the voxel selection operated by *t*-masking can, in principle, shorten the training time due to sparse active voxels and improve the DL model interpretability.

## Conclusion

In this work, we analyzed the application of a group-analysis-based FS, the *t*-masking, to deep neural network architecture, in the case of a very mild AD-vs.-NC classification task on a 180-example dataset of structural MR. We showed that the *t*-masking application could enhance the classification performance even better than could conventional FS techniques. Moreover, *t*-masking is generalizable to other binary classification tasks, different neuroimaging modalities, and other DL architectures.

## Data Availability Statement

Publicly available datasets were analyzed in this study. This data can be found here: https://www.oasis-brains.org (OASIS-3).

## Ethics Statement

The studies involving human participants were reviewed and approved by Comitato Etico IRCCS Pascale and OASIS-3 data sharing was granted by participants through informed consent and local IRB approval. The patients/participants provided their written informed consent to participate in this study.

## Author Contributions

DM: conceptualization, methodology, investigation, and writing–original draft. PB: methodology, writing, reviewing, and editing. PR and MS: writing, reviewing, editing, and supervision. MA: conceptualization, investigation, writing, reviewing, editing, and supervision. All authors contributed to the article and approved the submitted version.

## Conflict of Interest

The authors declare that the research was conducted in the absence of any commercial or financial relationships that could be construed as a potential conflict of interest.

## Footnotes

1 https://www.tensorflow.org/api_docs?hl=en
