# Supplementary figures and images for: Voxel-Wise Feature Selection Method for CNN Binary Classification of Neuroimaging Data
Source: Front Neurosci. 2021 Apr 20;15:630747. doi: 10.3389/fnins.2021.630747 (PMC8093438; doi:10.3389/fnins.2021.630747)

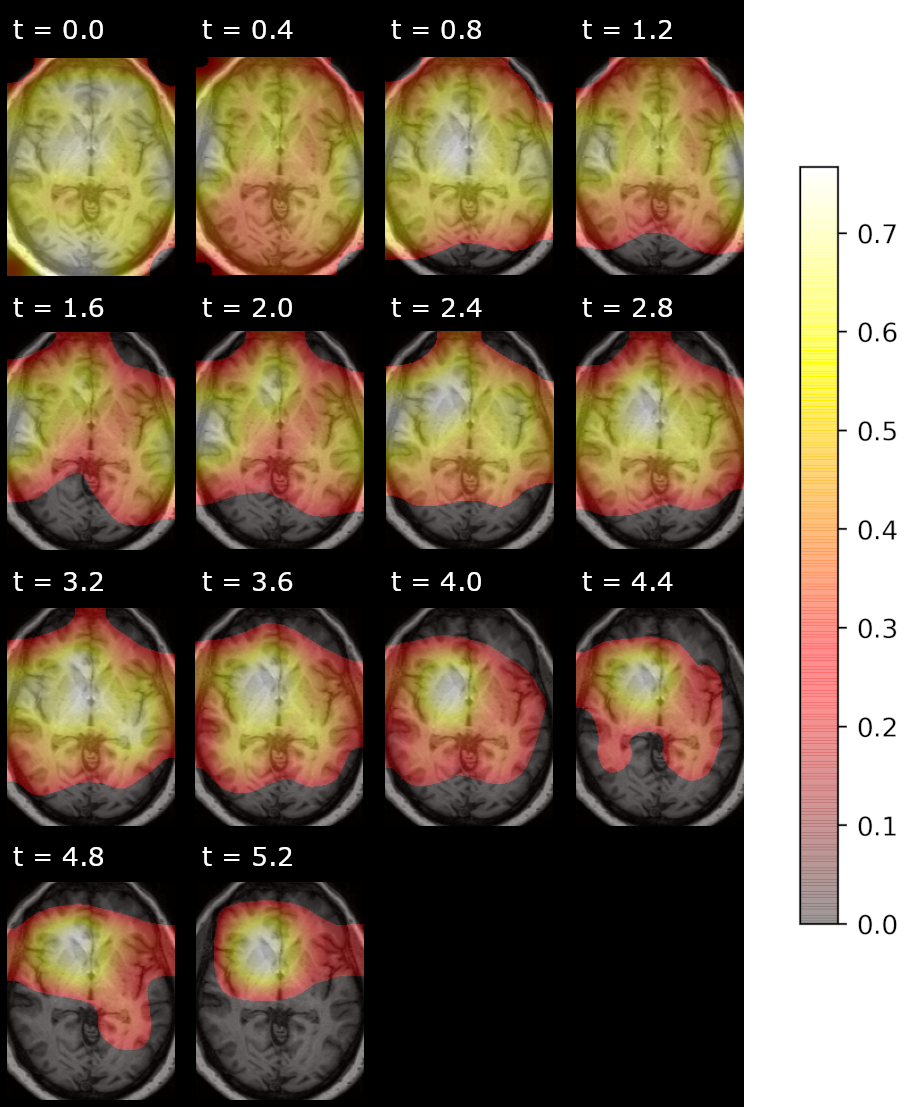

Supplement: Supplementary file 1 [file Image_1.TIF]
